# Unveiling OASIS family as a key player in hypoxia–ischemia cases induced by cocaine using generative adversarial networks

**DOI:** 10.1038/s41598-022-10772-1

**Published:** 2022-04-25

**Authors:** Kyoungmin Lee, Taehyeong Kim, Mookyung Cheon, Wookyung Yu

**Affiliations:** 1 Department of Brain Sciences, DGIST, Daegu, 42988 South Korea; 2 Dementia Research Group, Korean Brain Research Institute, Daegu, South Korea

**Keywords:** Machine learning, Gene regulatory networks, Gene expression, Reward

## Abstract

Repeated cocaine use poses many serious health risks to users. One of the risks is hypoxia and ischemia (HI). To restore the biological system against HI, complex biological mechanisms operate at the gene level. Despite the complexity of biological mechanisms, there are common denominator genes that play pivotal roles in various defense systems. Among these genes, the cAMP response element-binding (*Creb*) protein contributes not only to various aspects of drug-seeking behavior and drug reward, but also to protective mechanisms. However, it is still unclear which *Creb* members are key players in the protection of cocaine-induced HI conditions. Herein, using one of the state-of-the-art deep learning methods, the generative adversarial network, we revealed that the OASIS family, one of the *Creb* family, is a key player in various defense mechanisms such as angiogenesis and unfolded protein response against the HI state by unveiling hidden mRNA expression profiles. Furthermore, we identified mysterious kinases in the OASIS family and are able to explain why the prefrontal cortex and hippocampus are vulnerable to HI at the genetic level.

## Introduction

Cocaine addiction is one of the most popular drug abuses and leads to hypoxia–ischemia (HI) Daras, Tuchman^1^. HI is the third leading cause of death in the United States, yet unlike ischemic damage to other tissues, the degree of severity among damaged areas in the brain is not homogenous by region. For example, cocaine-induced vasoconstriction particularly jeopardizes the prefrontal cortex (PFC) and may contribute to compulsive cocaine intake^2^, and easily damages the hippocampus (HIP) area^3,4^. Many cocaine addictions result in different levels of mRNA levels in different regions of the brain^5–7^. Therefore, this selectivity can be explained, in part, by the diversity of mRNA expression profiles in each brain area. For example, upregulation of glial fibrillary acidic protein (*Gfap*) strongly indicates an activation of astrocytes following HI conditions^8^ and many past studies^9–11^ also show cocaine intake leads to significant upregulation of endothelin-1 (*Edn1*) which is a strong biomarker for vasoconstriction. Inevitably, this narrowed blood vein results in HI. During HI, hypoxia-inducible factor (HIF) accumulates^12^ and triggers regulation of many genes such as vascular endothelial growth factor (*Vegf*)^13^ and fibro blast growth factor (*Fgf*)^14^ which are heavily involved in angiogenesis, one of the famous defense mechanisms in response to HI.

Another famous defense mechanism is unfolded protein response (UPR) in response to endoplasmic reticulum (ER) stress caused by cocaine’s disturbance of calcium (Ca^2+^) and the influx of misfolded protein^15,16^. When ER stress occurs, the cell undergoes the following four reactions to overcome ER stress. (1) Reduction of the influx of new protein into ER by attenuating the translation, (2) enhancement of capability of protein folding, (3) ER stress-associated degradation (ERAD) that eliminating misfolded or unassembled protein from the ER and finally (4) the apoptosis, when the ER cannot be overcome by aforementioned three methods and cannot restore its function^16,17^. From the detection of ER stress to UPR, various genes are involved such as *eIF2a* and its four kinases and cAMP response element-binding protein (*Creb*)^18,19^. And other UPR markers such as Ern1(*Ire1*), *Atf6* and its downstream pathway genes, *Atf4, Ask1* and *Jnk*, play a vital role in this ER-associated defense mechanism^20^.

In addition, decades of research on cocaine addiction have revealed that several genes such as *deltaFosB*, *Dlg1*, *JunB*, and AMPA receptor subunit *GluR2*^21,22^, are implicated in cocaine addiction. Among these cocaine addiction-related genes, the cAMP response element-binding protein *(Creb*) has many subfamilies, such as *Creb1,2*, OASIS family (*Creb3, Creb3L1, Creb3L2, Creb3L4, Creb4*) and *Creb5,* and plays a pivotal role not only in the regulation of the rewarding effects of cocaine^23^ but also in protecting against oxidative stress^24^. However, it is still unclear which *Creb* members play an important protective role in cocaine-induced HI conditions. For example, hypoxia-inducible factor (HIF), in tandem with *Creb* mediates angiogenesis, one of the main responses to HI, remains elusive as to what kind of *Creb* binds to HIF. Another veiled mechanism of *Creb* under HI conditions is that the canonical kinases of *Creb* are no longer involved in the phosphorylation of *Creb*^25,26^.

Herein, to reveal high-temporal resolution on the expression of diverse gene that contributes to the multiple defense and restoring systems of the cells caused by chronic cocaine addiction, we set out on the unveiling of mRNA profile patterns using a cutting-edge deep learning method, generative adversarial networks (GAN) inspired by Park et al.^27^.

GAN is a class of machine learning developed by Goodfellow et al. in 2014^28^. It has two competing modules that are jointly optimized. The generator module G learns how to transform an input noise distribution p(z) into the underlying data distribution p_data_(x). The discriminator module D learns to distinguish between the empirical data x and the artificially generated G(z) data. G and D play a “game,” such that G aims to minimize, and D aims to maximize the objective function.

**Figure 1:**
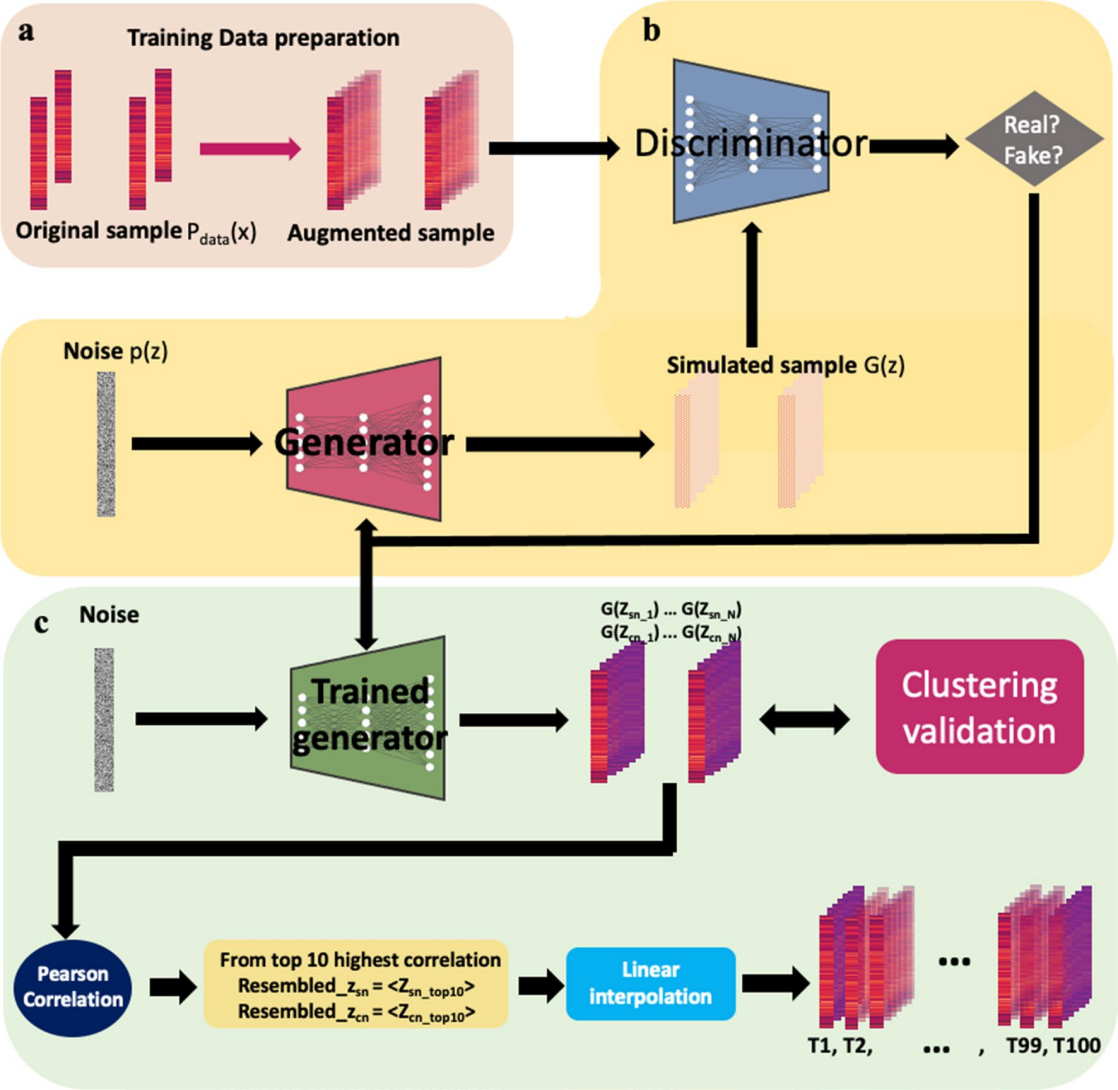
WGAN workflow. (**a**) Training Data preparation for GAN training. Simple tenfold linear augmentation method is applied. (**b**) GAN training framework structure (**c**) Generating fined time interval data using trained network. G represents generator. z is noise vector. T1–T100 represents time point from 1 to 100.

In vision area, GAN has shown superb a performance in that generated new data looks at least superficially authentic to human observer^29,30^. Hence, we apply this excellent capability of GAN in generating undistinguishable real-like data to bulk RNA-seq data. Using GAN, we generated the hidden mRNA expression pattern between the initial and final stages of the actual experiment, breaking the limit of bulk mRNA method that can only generate data from the beginning and the end of the experiment. A general overview of the GAN is displayed in Fig. 1. More details and general explanations for GAN are provided in the “Methods” section.

After applying the GAN to cocaine self-administration (SA) data^31^, we are able to found that the OASIS family, particularly *Creb3*, *Creb3l1*, and *Creb3l2*, are the key player genes in multiple defense mechanisms that respond to cocaine-induced stress states.

## Results

### HI induced by chronic cocaine SA

**Figure 2:**
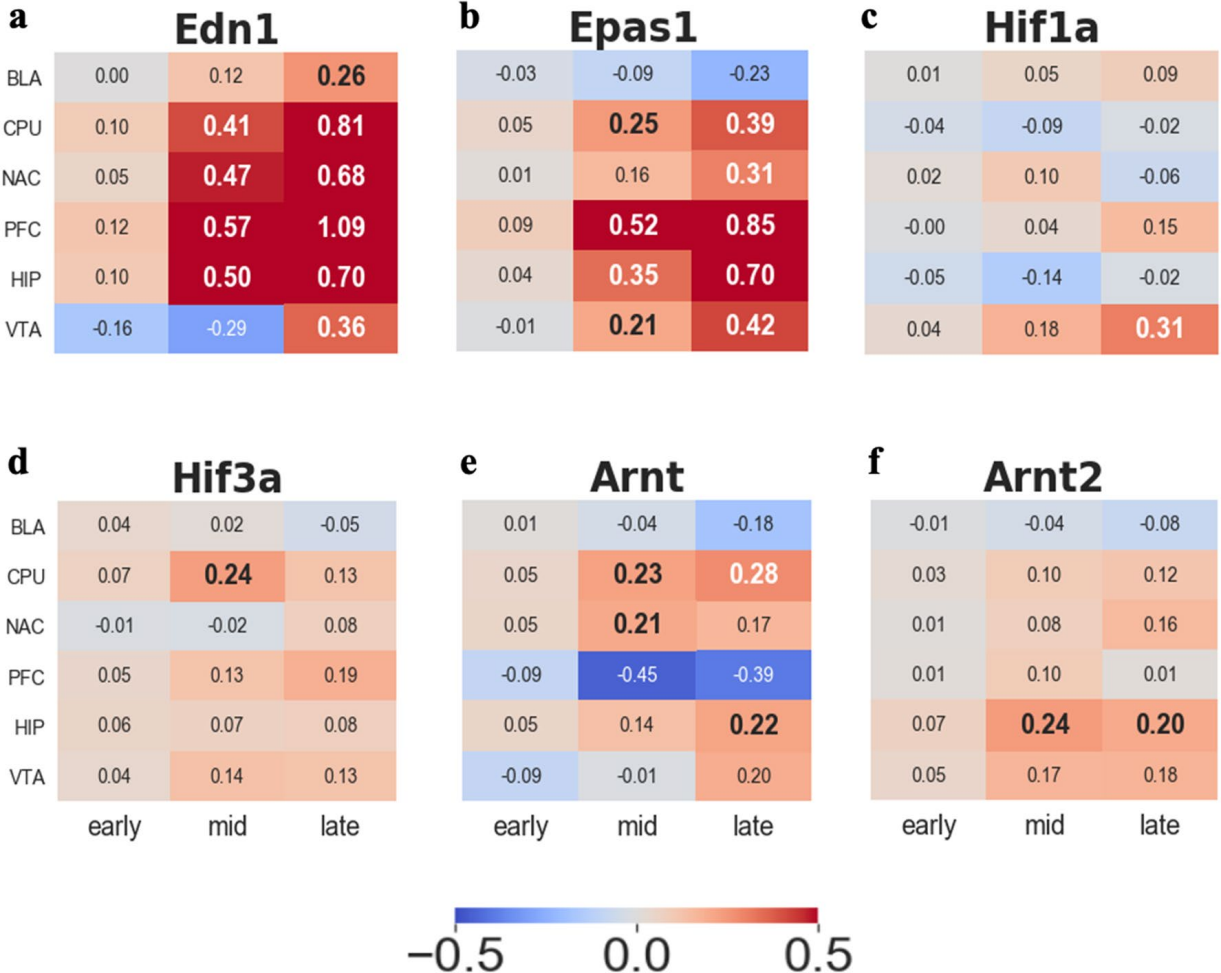
Log2FC trend for hypoxia-related genes. All the numbers in the heatmap indicates Log2FC trend value. (**a**) Endothelin1 (*Edn1*) (**b**) Endothelial PAS domain-containing protein 1 (*Epas1*) (**c**) Hypoxia-inducible factor 1-alpha (*Hif1a*) (**d**) Hypoxia-inducible factor 3-alpha (*Hif3a*) (**e**) aryl hydrocarbon receptor nuclear translocator (*Arnt*) (**f**) aryl hydrocarbon receptor nuclear translocator 2 (*Arnt2*).

HI is one of the repercussions of chronic cocaine addiction^32^. This is because cocaine increases the production of the potent vasoconstrictor *Edn1* mRNA^9,10^. Escalated levels of *Edn1* (Fig. 2a) lead to oxygen deprivation or lack of provision of nutrients, eventually ending up in HI conditions. To validate whether our data indicate hypoxia, we examined hypoxia biomarkers, the hypoxia-induced factor family (Fig. 2b–f). Normally, HIF 1 alpha subunit (Hif1-a) is a well-known biomarker of hypoxia^33–35^, but we have a significant upregulation of *Epas1* (Fig. 2b), indicating prolonged hypoxia, rather than *Hif1-a*, a transient hypoxia biomarker^36^ (Fig. 2c).

To adapt to adverse stimuli, such as cocaine-induced HI conditions, cells have diverse intrinsic defense and restoring mechanisms, such as UPR regulated by phosphorylation of *eIF2a* and angiogenesis^19,37^. Although a previous study reported the link between angiogenesis and ER stress^38^, the detailed mechanism has not yet been completely elucidated. Herein, in combination with our method and previous studies, we propose a big picture of the defense mechanism in terms of HI conditions caused by repetitive cocaine intake.

### Quality-control of ER in response to stress

Several studies have shown evidence of ER stress after HI. Cocaine increases intracellular calcium (Ca^2+^) concentration in the brain^39^, which leads to the disturbance of Ca^2+−^dependent protein folding and Ca^2+−^homeostasis within the ER. To control this stressed state, the ER engages the UPR, an adaptive response that mitigates unfolded protein accumulation to maintain cell function. One of the UPRs is the phosphorylation of *eIF2a*. By activating *eIF2a,* cells inhibit general translation initiation.

**Figure 3:**
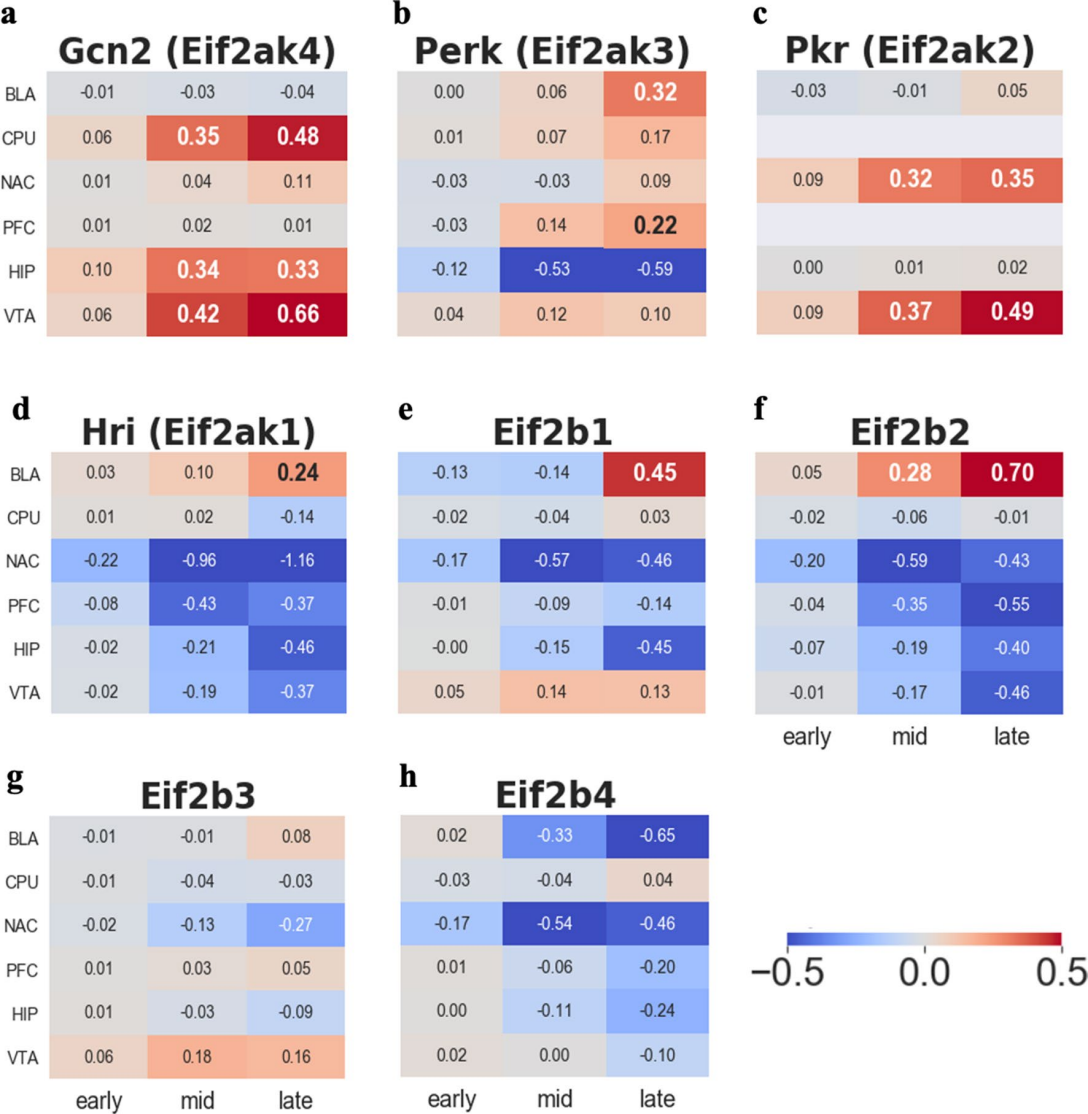
Log2FC trend for general translation inhibition related genes. (**a**) General control nonderepressible 2 (*Gcn2*) (**b**) PKR-like endoplasmic reticulum kinase (*Perk*) (**c**) protein kinase R (*Pkr*) (**d**) heme-regulated inhibitor kinase (*Hri*) (**e**) Translation initiation factor eIF-2B subunit alpha (*Eif2b1*) (**f**) Translation initiation factor eIF-2B subunit beta (*Eif2b2*) (**g**) Translation initiation factor eIF-2B subunit gamma (*Eif2b3*) (**h**) Translation initiation factor eIF-2B subunit delta (*Eif2b4*).

To activate *eIF2a*, one of the four kinases is required, heme-regulated inhibitor kinase (*Hri: Eif2ak1*), protein kinase R (*Pkr: Eif2ak2*), PKR-like endoplasmic reticulum kinase (*Perk: Eif2ak3*), and general control nonderepressible 2 (*Gcn2: Eif2ak4*). Our data showed a significant upregulation of *Gcn2* in the Caudate putamen (CPU), Hippocampus (HIP), and Ventral tegmental area (VTA) (Fig. 3a). *Gcn2* is activated by amino acid deprivation^40,41^. This implies that nutrient deprivation is inseparable from hypoxia during ischemia. *Perk*, one of the UPR markers, was upregulated in the BLA and PFC roughly after 10 days (beginning of the late time period) of the onset of cocaine administration, except in the HIP (Fig. 3b). The onset of upregulation in *Gcn2* is noticeable from mid-period, whereas an increase in *Perk* starts late, suggesting that sensing amino acid deprivation faster than activation of *Perk* or *Gcn2* outweighs *Perk* when it comes to inhibiting general translation in the HI condition. *Pkr* also shows the start of upregulation in the mid-period in the VTA and NAC (Fig. 3c), implying that the depletion of Ca^2+^ in the VTA and NAC occurs faster than in other regions of the brain^42^. Interestingly, unlike other kinases, *Hri* was significantly downregulated in all five regions of the brain, except the basolateral amygdala (BLA) (Fig. 3d). This is probably due to the common symptom of cocaine intake, which reduces nitric oxide (NO). Since *Hri* activation depends on NO binding^43,44^, this inhibition of *Hri* (Fig. 3d) is responsible for the conditions of HI. In addition, the overall reduction level of the *eif2b* family also strengthens the evidence that inhibition of general translation (Fig. 3e–h).

### OASIS family as an alternative quality control in response to ER stress caused by HI

**Figure 4:**
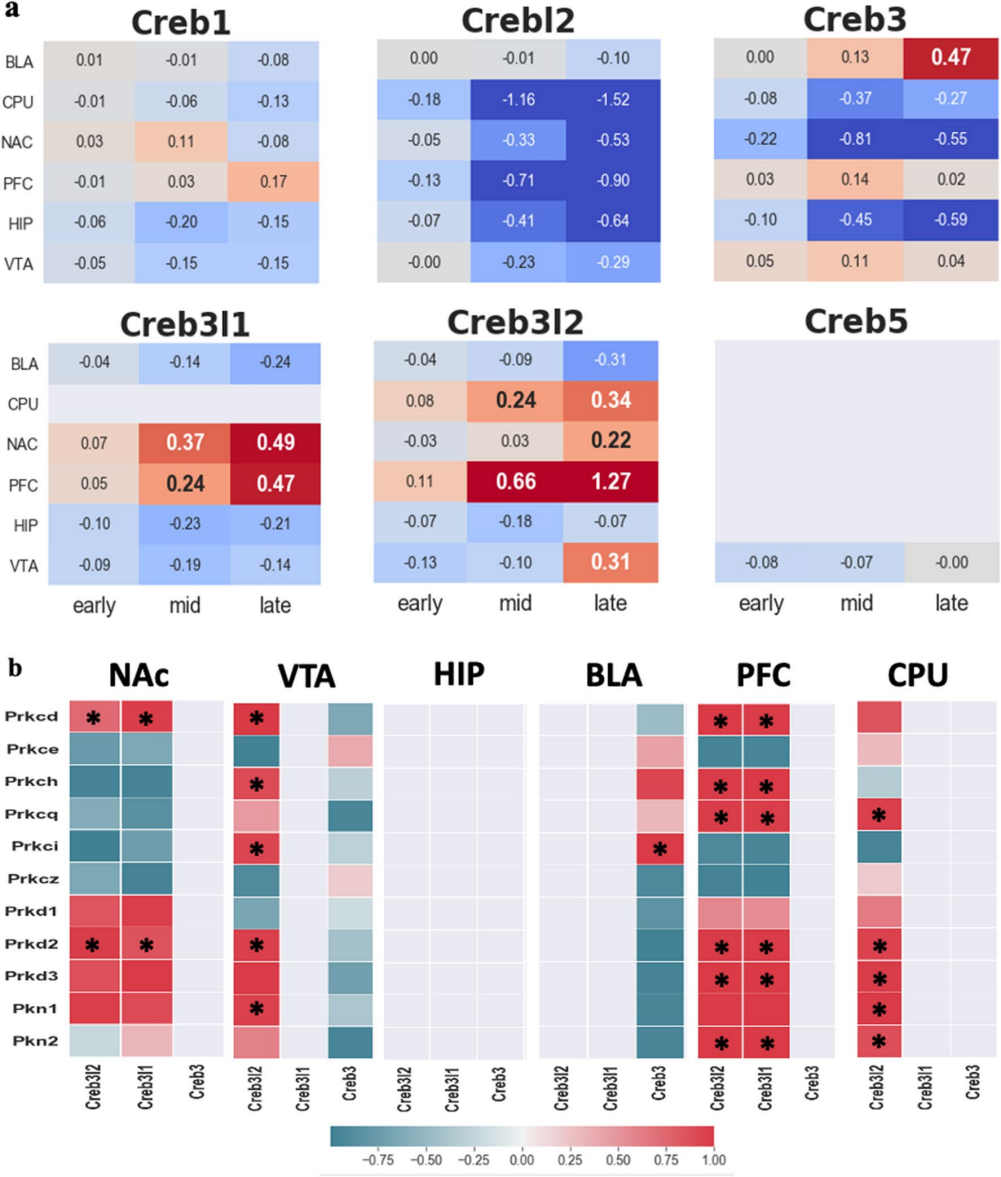
Log2FC trend for cAMP response element-binding protein (*Creb*) family and their coregulation with *Pkc* family. (**a**) Among *Creb* family, three of OASIS family shows upregulation pattern of Log2FC trend; *Creb3* increases significantly from late stage of BLA, *Creb3l1* shows upregulation from mid stage of NAC and the PFC and upregulation of *Creb3l2* occurs from mid stage of CPU and the PFC and late stage of NAC and VTA, respectively. (**b**) Coregulation between *Pkc* family and OASIS family. Colored rectangles indicate only upregulation gene. Asterisk *indicates differentially expressed genes (Log2FC > 0.2) on both rows and columns. Reddish color indicates positive correlation between the genes. Teal color indicates negative correlation.

Despite the increase in the two canonical UPR marker profiles, *Perk* and *Ern1*(*Ire1),* the other canonical UPR marker, *Atf6*, and their downstream pathways, such as *Atf4*, *Ask1*, *Jnk*, and *Hspa5*, are strongly suppressed in many regions of the brain. (Supplementary Figs. 3c–g, 4i). A previous study also mentioned the discordance of these canonical UPR pathways that are not activated concurrently in the HI brain^45^. Due to incomplete responses to ER stress, other ER transducers, such as the OASIS family, can compensate for the canonical branch of the UPR^46^. Our data show increased *Creb3* levels were found in the BLA (Fig. 4a), leading to an enhancement of cellular tolerance to ER stress^46^. Similar to a previous study^47^ that used an ER stress inducer, our data showed the increment level of *Creb3l1* in the NAC and PFC (Fig. 4a). Elevated levels of *Creb3l2* were also found in four regions of the brain, except for the BLA and HIP (Fig. 4a). The onset of the increment of *Creb3l2* started at mid (around 4 days after the onset of cocaine SA), implying that the late phase of UPR occurred around the mid-period^48^.

To activate *Creb*, kinases are required. However, a previous study claimed that under the HI condition *Creb* was not mediated by any Ca^2+^-dependent kinases such as conventional protein kinase C (*Pkc*), Ca^2+^/calmodulin-dependent protein kinase (*CaMK*) and other canonical *Creb*’s kinase *Pka* and ribosomal protein S6 kinase (*Rsk*) family^25^. Hence, to select potential candidate kinases for *Creb* under HI conditions, we narrowed the range of candidates down to three kinases, the novel protein kinase C (*nPkc*) family, protein kinase D (*Pkd*) family, and protein kinase N (*Pkn*). This malfunction of Ca^2+^-dependent kinases may be attributed to Ca^2+^ depletion events that occur in the ER or mitochondria^49^ under HI conditions. Therefore, Ca^2+^ independent *Creb* kinases, such as *nPkc*, *Pkd*, and *Pkn*, can be potential candidates for activating *Creb*. Of *nPkc*, *Pkc*-δ (*Prkcd*), *Pkc*-η (*Prkch*), and *Pkc-θ* (*Prkcq*) have the most similar patterns of transcriptome trajectories as *Creb3l1* and *Creb3l2* in many areas of the brain (Fig. 4b). Among the atypical *Pkc* families, *Pkc*-*I* (*Prkci*) and *Pkc- ζ (Prkcz)*, only *Pkc*-*ι* (*Prkci*) has a positive correlation with *Creb3l2* and *Creb3* in the VTA and BLA areas, respectively (Fig. 4b). *Pkc-ι* is known for preventing amyloid-beta-induced apoptosis^50^. For the *Pkd* family, *Pkd2* (*Prkd2*) shows positive correlations in all areas of the brain, except the HIP and BLA areas, and *Pkd3*(*Prkd3*) has positive correlations in PFC and CPU, and compelling upregulation in the PFC area (Fig. 4 and Supplementary Fig. 5i). The reason why the *nPkc* family shows a strong correlation pattern with *Creb3l1* and *Creb3l2* might be that, unlike the conventional *Pkc* family, they require only diacylglycerol (DAG) instead of Ca^2+^. The same applies to *Pkd*. For the atypical *Pkc* family, neither DAG nor Ca^2+^ is required.

### Angiogenesis in the cocaine-induced HI condition

**Figure 5:**
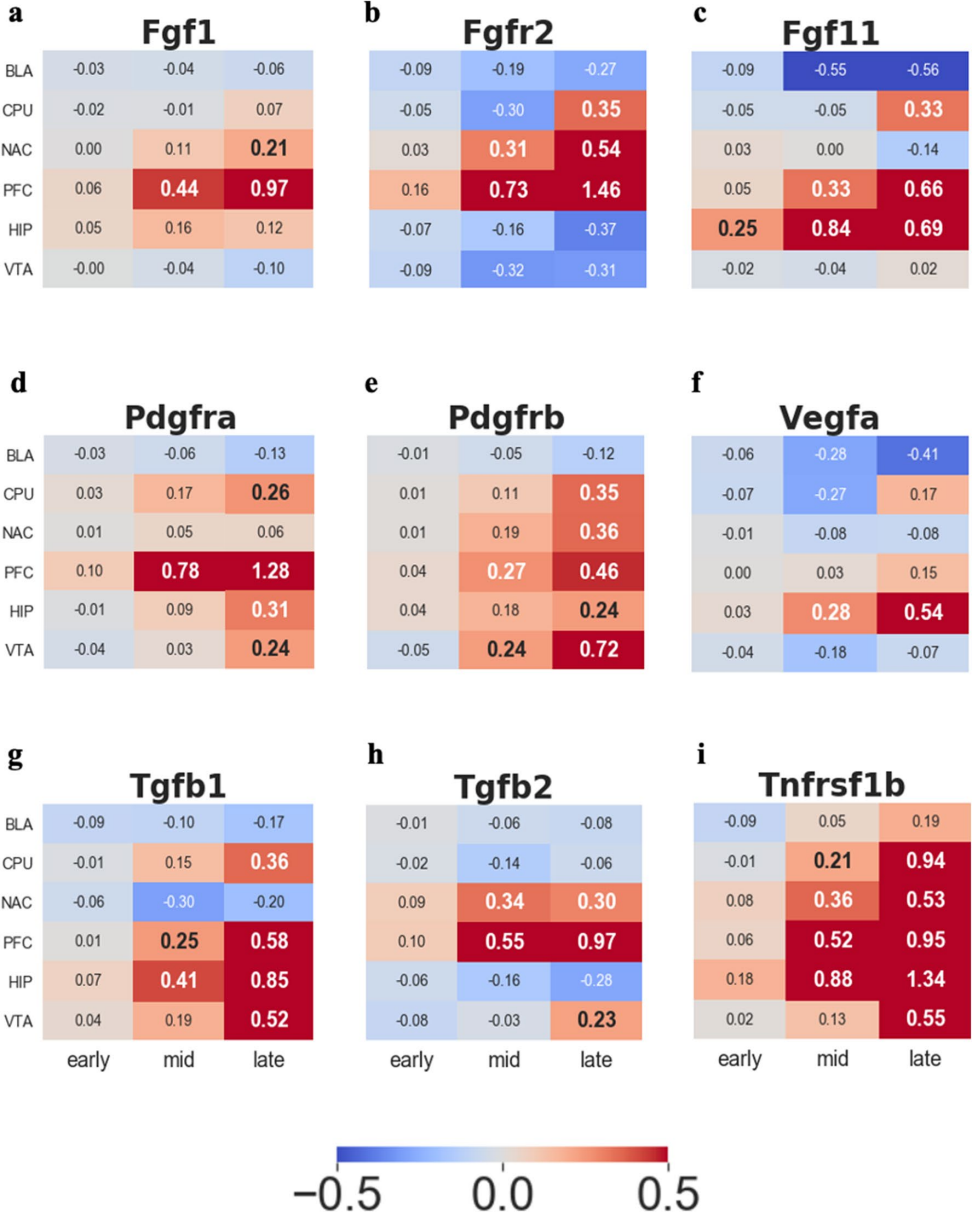
Log2FC trend for angiogenesis-related genes. (**a**) Fibroblast growth factor 1 (*Fgf1*) (**b**) Fibroblast growth factor receptor 2 (*Fgfr2*) (**c)** Fibroblast growth factor 11 (*Fgf11*) (**d**) Platelet-derived growth factor receptor A (*Pdgfra*) (**e**) Platelet-derived growth factor receptor A (*Pdgfrb*) (**f**) Vascular endothelial growth factor A (*Vegfa*) (**g**) Transforming growth factor beta 1 (*Tgfb1*) (**h**) Transforming growth factor beta 2 (*Tgfb2*) (**i**) Tumor necrosis factor receptor 2 (*Tnfr2*).

Angiogenesis is a well-known mechanism that responds to HI conditions. A great deal of previous studies shows the occurrence of angiogenesis as a repairing system in response to the HI condition^51,52^. The combination of chronic cocaine addiction and HI conditions proposes the indication of angiogenesis since angiogenesis-related genes, such as vascular endothelial growth factor (*Vegf*)^53^, transforming growth factor-beta (*Tgfb*)^54^, tumor necrosis factor receptor superfamily (*Tnfrsf1b*)^55^, platelet-derived growth factor (*Pdgfr*)^56^ and fibro blast growth factor (*Fgf* and *Fgfr*)^57^, showed significant upregulation started mid-period in many areas of the brain (Fig. 5). In our data, *Fgf* was more upregulated than *Vegf* because *it* is a more potent angiogenic factor than *Vegf*^58^.

**Figure 6:**
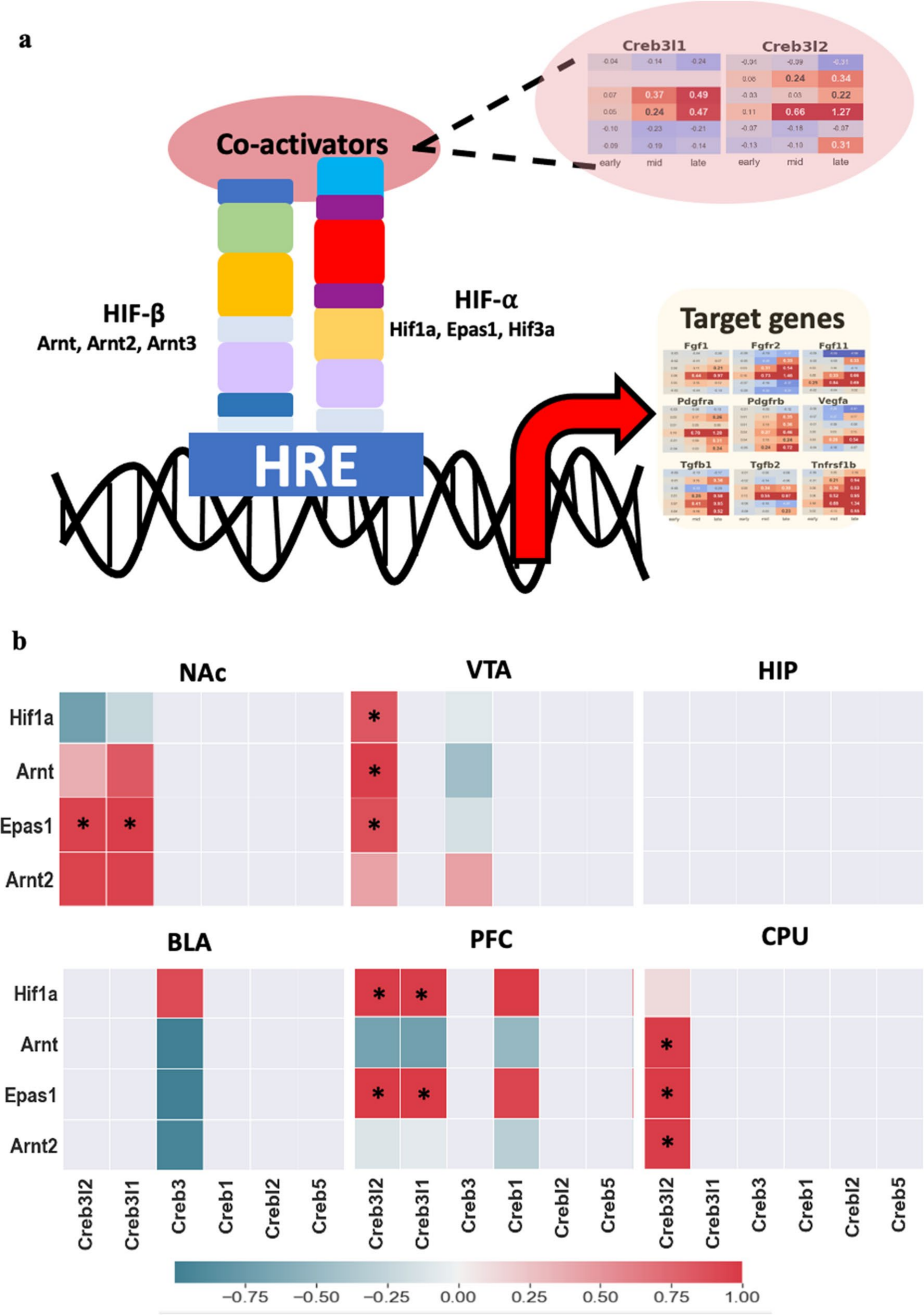
Member of OASIS family as co-activators for HIF family. (**a**) Description of *Creb3l1* and *Creb3l2* as co-activator with HIF family for mediating angiogenesis. (**b**) Coregulation between HIF and OASIS family. Colored rectangles indicate only upregulation gene. Asterisk *indicates differentially expressed genes (Log2FC > 0.2) on both rows and columns. Reddish color indicates positive correlation between the genes. Teal color indicates negative correlation.

Angiogenesis is mediated by the HIF family^59^. HIF binds to the hypoxia response element (HRE) and regulates its target genes, such as angiogenesis-related genes (Fig. 6a). Interestingly, phosphorylated *Creb* also binds to HRE^60^ and both HIF and *Creb* interact with the *Creb* binding protein (*Cbp*). This indicates that *Creb* can be a potent mediator of HIF in angiogenesis. However, it is still unclear which *Creb* family can be a potential cofactor for HRE. Our method revealed that *Creb3l1* and *Creb3l2* are strong candidates for the cofactor of HRE (Fig. 6b). In the HIF family, *Epas1* showed strong coregulation with *Creb3l1* and *Creb3l2* in four areas of the brain, except the HIP and BLA (Fig. 6b). This could be the habitation of *Creb3l1*, *Creb3l2* and *Epas1* are overlapped. Unlike *Hif1a*, which is ubiquitously expressed, one of the main residences of *Epas1* is astrocytes and endothelial cells^51^, and so are *Creb3l1* and *Creb3l2*^47^. This result supports the idea that astrocytes can modulate angiogenesis^61^. Interestingly, the significant upregulation of *Gfap* (Supplementary Fig. 4a), the best biomarker for reactive astrocytes following injury or stress in the central nervous system^62^, indicates the involvement of astrocytes in HI conditions. Therefore, it is conceivable that *Creb3l1* and *Creb3l2* with *Epas1* could be key players in angiogenesis.

### Other defense mechanisms in the HI condition

In addition to the role of the *Creb3* family in mediating angiogenesis, its own repairing role of *Creb3l1* and *Creb3l2* also contributes to the repair system. Upregulation of *Creb3l1* was found proximal to the injury site, and *Creb3l2* was also found in the peri-infarction region in a brain ischemia mouse model to restore the injury region^47^. Our data showed a significant *Creb3l2* increase in four areas of the brain except for the BLA and HIP, and *Creb3l1* increased in the NAC and PFC (Fig. 4a).

Other signals, such as upregulation of *Pkd*, *Pkn*, and the novel *Pkc* family (Supplementary Fig. 5) might imply regulation of angiogenesis and proteolytic as a defense or repair mechanism^63–65^.

### Spatial differences of the defense system responding to HI condition

Despite the general upregulation trend of the transcriptome patterns of our selected genes in six areas of the brain, the transcriptome profile showed spatial differences between the brain areas. For example, HI-related biomarkers, *Edn1*, *Epas1*, and *Pkc-δ,* have a striking upregulation pattern in the PFC, intensified after the mid-period and genes that play a role in the repair system, and *Gfap* also soared dramatically in the PFC (Fig. 2a,b and Supplementary Figs. 4a, 5a). Since blood vessel are strongly constricted by *Edn1*^9^, oxygen concentration fall causes an increase in *Epas1*, and also *Gfap* is induced by HI^8^, these results provide evidence that PFC were attacked intensively.

In our data, the HIP also showed a general upregulation trend of angiogenesis-related genes (Fig. 5), and a significant increase in *Gfap* began in the mid-period (Supplementary Fig. 4a), which is circumstantial evidence of damage. This might indicate why the HIP is particularly vulnerable to HI^66^. Our results also suggest possible explanations for this defenseless of the HIP to HI. None of the OASIS family members was upregulated in the HIP region (Fig. 4).

Lastly, among our selected genes, a relatively opposite transcriptome pattern tendency occurred in BLA compared to other brain regions such as *Epas1*, *Arnt1,2, Eif2b*, *Creb3*, and angiogenesis-related genes (Figs. 2, 3, 4a, 5). This might indicate that BLA is relatively intact in HI compared to other brain regions. One possible reason for the BLA intactness hypothesis is the upregulation of *Creb3* (Fig. 4a) since *Creb3* potentiates cellular tolerance^46^.

**Figure 7:**
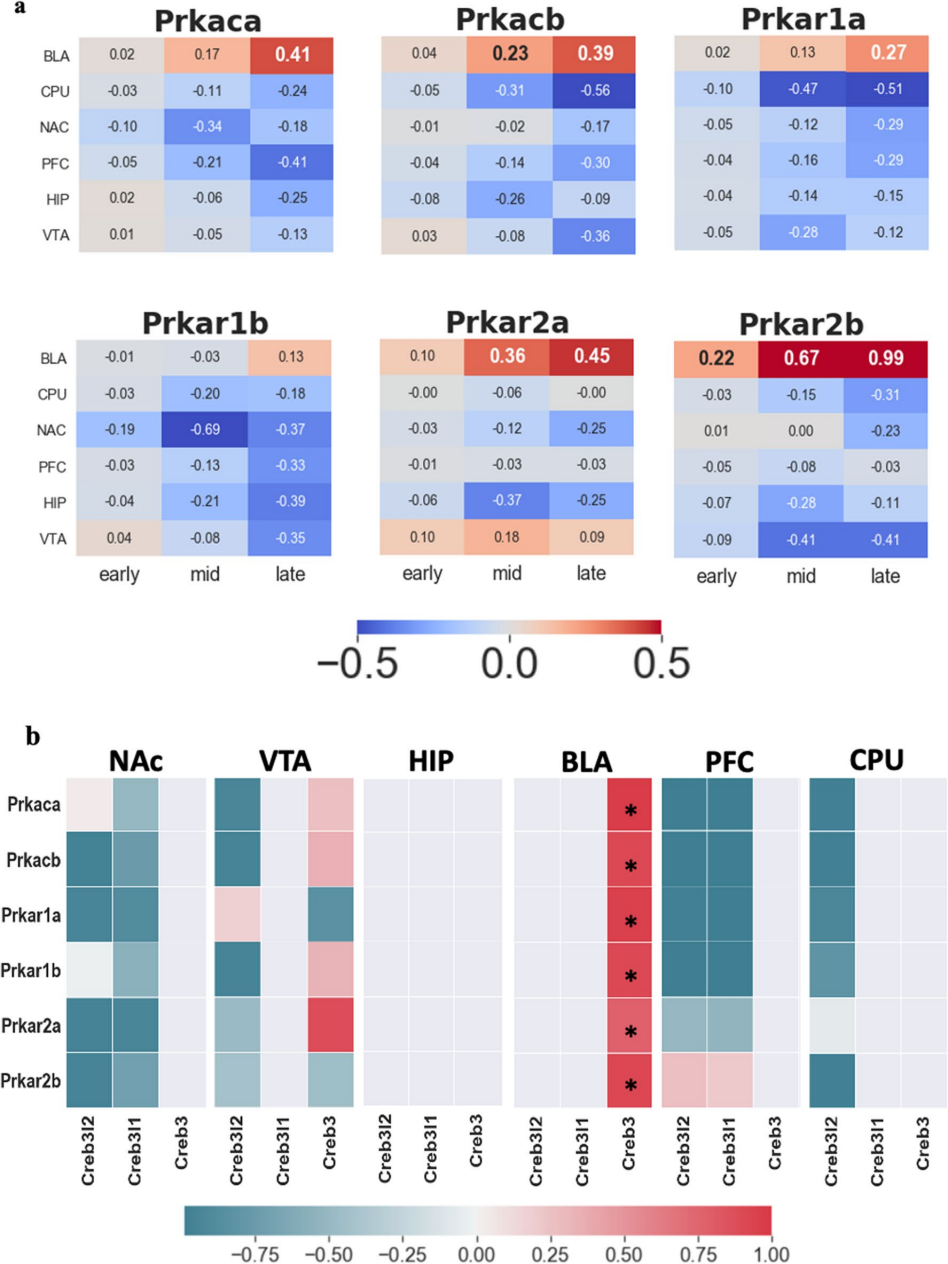
Relationship between *Pka* family and OASIS family. (**a**) Log2FC trend for *Pka* family in six brain regions. (**b**) Coregulation between *Pka* and OASIS family. Colored rectangles indicate only upregulation gene. Asterisk *indicates differentially expressed genes (Log2FC > 0.2) on both rows and columns. Reddish color indicates positive correlation between the genes. Teal color indicates negative correlation.

Another intriguing result is the increased level of *Pka*, which was only found in the BLA (Fig. 7a). And only *Creb3* among the *Creb* family shows strong association with *Pka* (Fig. 7b).

## Discussion

The level of transcriptome unveiled by our method sheds light on more detailed knowledge about the association between chronic cocaine addiction and HI conditions from four perspectives: (1) Heterogeneous expression patterns of *eIF2a* kinases in response to cocaine-related stress; (2) The OASIS family is an alternative candidate to compensate for the incomplete canonical UPR mechanism and reveal its mysterious kinase candidates; (3) The OASIS family is a cofactor with HIF for mediating angiogenesis and various defense mechanisms in response to HI conditions; and (4) Spatially different mRNA levels in response to cocaine-induced HI conditions.

**Figure 8:**
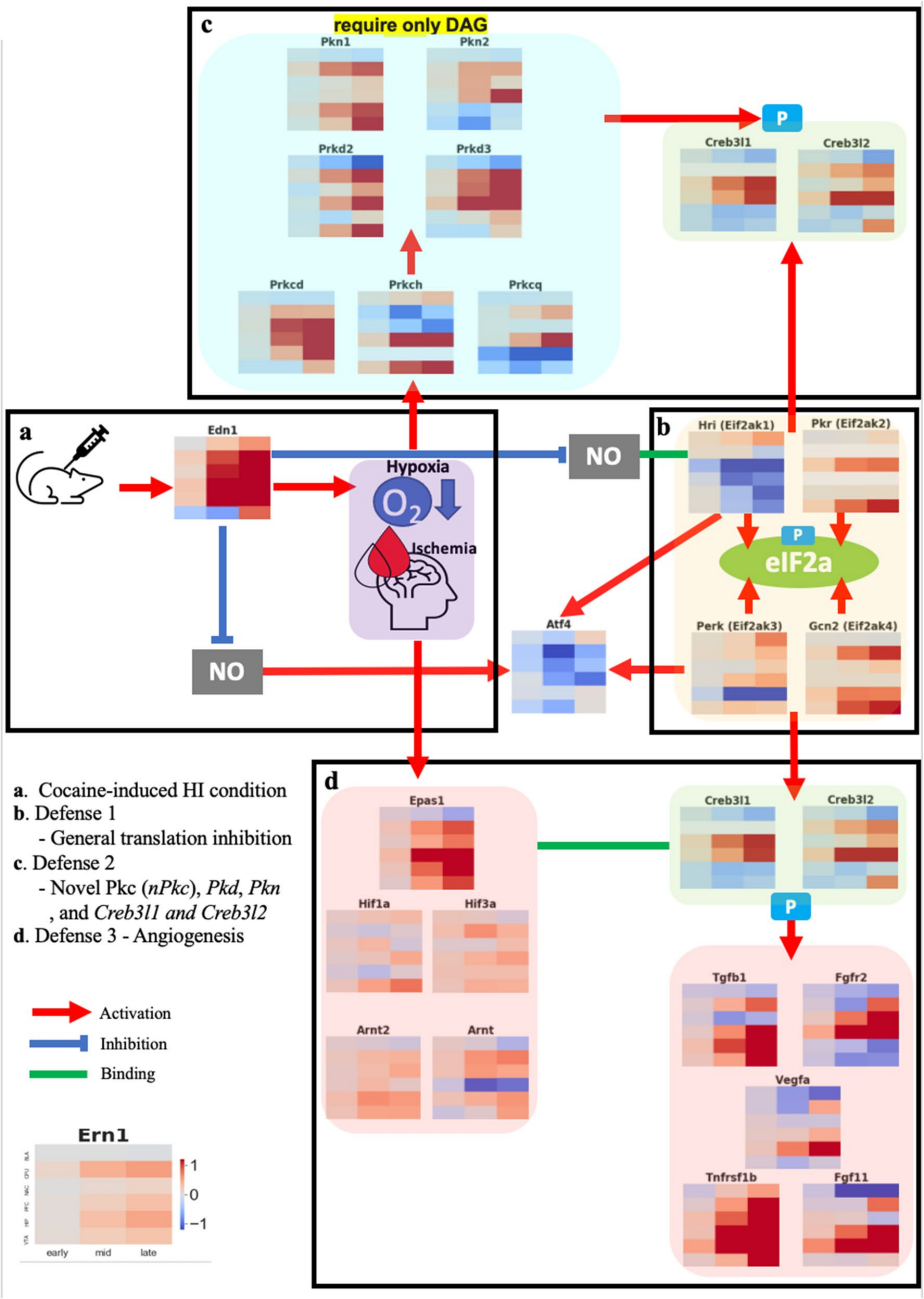
Overall defense mechanism in HI condition. (**a**) Cocaine causes upregulation of *Edn1* which leads to inhibition of nitric oxide (NO) and hypoxia–ischemia (HI). (**b**) Phosphorylation of *eIF2a* caused by its kinases leads to inhibition of general translation (**c**) *nPkc, Pkd* and *Pkn* family function as not only defense system against HI but also phosphorylated *Creb3l1* and *Creb3l2*. (**d**) HIF interacts with *Creb3l1* and *Creb3l2* and they mediate angiogenesis.

*p-eIF2a*, an indicator of the inhibition of general translation, is normally accompanied by upregulation of *Atf4*. Many previous studies have reported a significant relationship between *p-eIF2a* and *Atf4* ^67,68^. Especially, *Perk-p-eIF2a − Atf4* is a well-known pathway involved in ER stress. However, strikingly, our results showed inhibition of *Atf4* (Supplementary Fig. 3c) because the prolonged HI condition resulted in incomplete UPR results, such as significant inhibition of *Atf4* in all six regions of the brain (Supplementary Fig. 3c). This inhibition might attribute to the strong inhibition of *Hri* (Figs. 3d, 8b), which is another activator of *Atf4*. Given the contrary profiling pattern between *Perk* and *Hri*, which both have an impact on *Atf4*, the present results can be interpreted as *Hri* somehow outweighs *Perk’*s impact on *ATF4*, at least in the prolonged hypoxia caused by chronic cocaine addiction. Furthermore, among the kinases of *eif2a*, *Gcn2,* and *Pkr* showed stronger upregulation than other kinases (Fig. 3a,c). This implies that nutrition deprivation and Ca^2+^ might be key factors for the inhibition of general translation in HI conditions induced by cocaine administration.

Among the canonical UPR markers, *Perk* and *Ire1* show an incremental trend in many regions of the brain, but *Atf6* has a decremental trend in five regions of the brain except the VTA. In addition to this disharmony of the canonical UPR pathway, its downstream signaling pathways lead to the same results, inhibition of apoptosis and autophagy. Supplementary Figs. 3 and 4 show the ER stress transducer, apoptosis, and autophagy-related genes. For example, increased levels of *Atf5* and *Mcl1* induced by *Creb3l1* and *Creb3l2* suggest inhibition of apoptosis (Supplementary Fig. 3h,i). The decreased levels of the *Atg* family, *Gabarapl1*, *Map3k5*, and *Mapk8,9,10* indicate strong inhibition of autophagy (Supplementary Figs. 3e–g, 4d–h and 6 shows an overview of the mechanism.

Normally, *Perk*, *Ire1*, and *Atf6* and their downstream signals respond in concert to attenuate the effects of ER stress. A previous study also reported incomplete UPR in the hypoxia-reperfusion case^45^. Instead of these canonical UPR transducers, noncanonical UPR transducers, *Creb3*, *Creb3l1*, and *Creb3l2,* have noticeable profile patterns in our data. This substitution of noncanonical UPR transducers for incomplete canonical UPR factors is still elusive under HI conditions, and thus it would be worthwhile to proceed further investigation. Interestingly, both inhibition of *Atf4* and activation of *Creb3l2* also lead to prevention of apoptosis in addition to inhibition of downstream of the canonical UPR transducer^69,70^. From this inhibition of apoptosis, we cannot exclude the possibility of tumorigenesis since impaired apoptosis is a prerequisite for tumorigenesis.

In response to cocaine-induced stress, there are various defense or repair mechanisms. We shed light on the repairing function of the OASIS family and its kinase calcium-independent *Pkc* family in HI conditions, as well as the role of the OASIS family as a potential co-activator with HIF for angiogenesis.

Interestingly, our findings, such as the OASIS family and *Epas1*, which are mainly detected in astrocytes, might imply the involvement of astrocytes in HI conditions. To support this idea, the significant upregulation of *Gfap* (Supplementary Fig. 4a), the best biomarker for reactive astrocytes^71^, indicates that astrocytes are deeply involved in HI conditions.

By elucidating high-resolution time points, we were also able to identify why the PFC and HIP are vulnerable to HI conditions and the possibility of intactness of BLA compared to other regions. The PFC and HIP showed us not only significant upregulation of HI marker genes but also angiogenesis-related genes. Especially, radical fluctuation of HI-related genes in PFC may be a possible explanation for why drug addicts would have trouble in suppressing their craving for drugs. This is because the PFC is known to be a control center for behavior and the damaged PFC can lead to impulsive action^72^. In BLA, our selected genes had a somewhat opposite pattern compared to other regions. This is probably due, in part, to the significant upregulation of the *Creb3* level, which gives cellular tolerance in response to stress. Interestingly, the canonical *Creb’s* kinase, the *Pka* family, shows a compelling upregulation trend and a strikingly high coregulation pattern with *Creb3* only in the BLA. BLA plays a pivotal role in condition-cue memory consolidation^73^ and its lesions abolish the ability of drug-paired stimuli to reinstate lever-pressing behavior for cocaine^74,75^. This is because cocaine affects the dopaminergic (DA) system, and the combination of long-term depression (LTD) with DA changes LTD to long-term potentiation (LTP), which is called dopamine-dependent plasticity^76^. Memory consolidation occurs via the LTP. One of the main switches required for this type of transition is *Pka*^76^. Therefore, we suggest that *Pka* might be involved in memory-associated roles with *Creb3* rather than a protective role, at least in cocaine-induced HI conditions. This supports the idea that dopamine-dependent plasticity requires *Pka* for the transition from LTD to LTP^76^.

In summary, Fig. 8 shows the overall defense mechanism against HIs induced by cocaine administration. Cocaine leads to elevated levels of *Edn1*, which then causes HI (Fig. 8a). HI causes Ca^2+^-depleted ER, which causes ER stress, and the inhibition of general translation occurs by *eIF2a* phosphorylation (Fig. 8b). The OASIS family, induced by ER stress, attempts to restore damaged cells (Fig. 8c) and plays a key role in mediating angiogenesis with HIF (Fig. 8d).

Lastly, GAN generated real-like data which was clustered perfectly very well with original data. Since applying deep learning method to biological data is not easy due to requirement of tremendous amount of data, hence GAN method is particularly advantageous for biological data that is inherently experiencing data shortage. For instance, applying GAN to brain-related or rare disease omics data which have fewer samples (tens to hundreds of samples) leads to effectively extract hidden patterns from the data and by unveiling this hidden information from the data can be deepened our understanding of molecular mechanism.

## Materials and methods

### Cocaine SA dataset

We used a public dataset generated by a previous study^31^ and followed their RNA-seq analysis method. We started our analysis from male C57BL/6 J mice bulk-mRNA seq count data, which were provided by the authors after a request by email. Mice were trained initially (3–10 d) for food reinforcement before cocaine self-administration using either active lever or inactive lever. Responding on active lever was reinforced on a fixed-ratio one (FR1) schedule and on the inactive lever results in no programmed consequence. Once the mice met acclimated to FR1 schedule, they were moved onto an FR5 schedule. For cocaine self-administration, FR1 resulted in a single (0.03 ml) infusion of cocaine (0.5 mg/kg/infusion over 3.5 s. Mice underwent 2 h daily session for 10–15 d:5–10 d on an FR1 schedule followed by 4–5 d of FR2 schedule. More detailed information is in the past study^31^.The dataset consists of transcriptome profiles from six regions of the brain: the basolateral amygdala (BLA), dorsal striatum (CPU), nucleus accumbens (NAC), PFC, HIP, and ventral tegmental area (VTA). It has 230 samples that have three to six replicas per region. We used six types of sample data classified by experimental design as follows: SN (saline SA + 24 h withdrawal), CN; chronic cocaine case (cocaine SA + 24 h withdrawal), SC (saline SA+30 d withdrawal+acute cocaine challenge), CS (cocaine SA+30 d withdrawal+acute saline challenge), CC (cocaine SA+30 d withdrawal+acute cocaine challenge in re-exposure context), and SS (saline SA+30 d withdrawal+acute saline challenge). The duration of cocaine SA is 10 − 15 days, which is considered chronic cocaine^77^.

### Normalization of count data for training

From the count data, we calculated reads per kilobase per million mapped reads (RPKM) using edgeR and the biomaRt library. We cut off any genes below RPKM 1 and maintained at least 80% of the samples per group. Then, we normalize the RPKM data considering the count size using the voom library from R for down analysis.

### Data augmentation and its validation

Because the original data has only three to eight samples, we adopted a data augmentation method that is prevalent in computer vision applications^78^. First, we performed a pairwise combination for each brain region per condition. For example, the CN condition in the BLA region has six original samples; hence, _6_C_2_ is 15 combinations made of original samples. Then, each pair in combination is augmented by a linear interpolation (S_aug_ = xS1 + (1 − x) S2, where x = (0.1, 0.2,…, 0.9) and S1 and S2 are the original samples in pairs under combination). For example, 9 × 15 = 135 augmented samples were generated in CN condition of the BLA. Thus, 6543 augmented samples were produced. We can now use a total of 6773 samples (230 original samples + 6543 augmented samples). They were rescaled using MinMaxScaler in the scikit-learn package of Python, which provides a computational advantage^79^. To validate the augmented data, we adopted the t-distributed stochastic neighbor embedding (t-SNE) method. The parameters were set as follows: n_components = 2; verbose = 1; perplexity = 40; and n_iter = 7000 (Supplementary Fig. 1).

### Wasserstein GANs with gradient penalty loss

We used the Wasserstein GAN and gradient penalty loss (WGAN-GP) for model training and the RMSprop optimizer. After generating the fake data, we filtered the data only over the Pearson correlation value of 0.95. We have documented other hyperparameters in our study. (Supplementary Table 1).

### Network architecture and initialization

We used fully connected the generator and the discriminator and their number of nodes are 800 and 400 respectively. For initializing weight, we randomly generated withing the range [− 0.3, 0.3] and for generating random distribution for latent space, we used the combination of Gaussian and Poission distribution.

### Averaged gene expression simulation

To generate resembled fakes, which is the average z of each sample, we generated 35,000 fakes using a trained generator (G) and picked 10 nearest latent vectors based on the Pearson’s correlation value between G(z) and the real sample.

The delta vector (δ) was created by subtracting the control (SN) from chronic cocaine-addicted state (CN). Then, we generated each gene expression level depending on the time point G(z_i_). The variable z_i_ is constructed using two variables as follows: z_i_ = z_SN_ + δ × i/100 (i is 0–99). This process creates a transition curve of gene expression from the normal state to the addictive state. We created an averaged gene expression profile by repeating this process ten times at different epochs (10–30 k) and the average gene expression transition curve (Supplementary Fig. 2).

To inspect temporally distinguished gene expression trends during cocaine addiction, we divided the entire time period into three-time intervals: early (1–34), mid (34–67), and late (67–100), and then calculated the geometric mean for each time interval, as each gene was not independent of each other. We then compared the gene expression level of each time frame using the log2 fold change.

## Supplementary Information

Supplementary Information.
