# Supplementary material for: Unveiling OASIS family as a key player in hypoxia–ischemia cases induced by cocaine using generative adversarial networks
Source: Sci Rep. 2022 Apr 25;12:6734. doi: 10.1038/s41598-022-10772-1 (PMC9038918; doi:10.1038/s41598-022-10772-1)
Supplement: Supplementary file 1 — Supplementary Information. [file 41598_2022_10772_MOESM1_ESM.pdf]

Unveiling OASIS family as a key player in hypoxia-ischemia cases in duced by cocaine using generative adversarial networks

Kyoungmin Lee[1,+], Taehyeong Kim[1,+], Mookyung Cheon[2*] and Wookyung Yu[1*]

[1]Brain and Cognitive Sciences, DGIST, Daegu42988, South Korea

[2]Dementia Research Group, Korean Brain Research Institute, Daegu, South Korea

*Correspondence: mkcheon@kbri.re.kr, and wkyu@dgist.ac.kr

[+]These authors contributed equally to this work.

Author contributions

K. L., T. K., M. C, and W-K.Y. designed the study. K. L. and T.K. performed the experiments. K. L., T. K., M. C, and W-K.Y. interpreted the data. K. L., T. K., M. C, and W-K.Y. wrote the manuscript. All authors contributed to the manuscript and approved the submitted version.

Keywords: Cocaine addiction, Generative Adversarial Network, OASIS family, hypoxia-ischemia

Supplementary Figure 1

a

Regions / Conditions	BLA	CPU	HIP	VTA	PFC	NAC
**CC**	6	7	5	5	7	7
**SS**	7	7	6	6	7	6
**SC**	7	6	7	5	7	5
**CS**	5	6	6	3	5	5
**CN**	6	8	8	6	8	8
**SN**	7	8	7	6	7	8

Linear Interpolation

Regions / Conditions	BLA	CPU	HIP	VTA	PFC	NAC
**CC**	141	196	95	90	196	196
**SS**	196	196	141	141	196	141
**SC**	196	141	196	95	196	95
**CS**	95	141	141	30	95	95
**CN**	141	332	332	141	332	332
**SN**	196	332	196	141	196	332

b

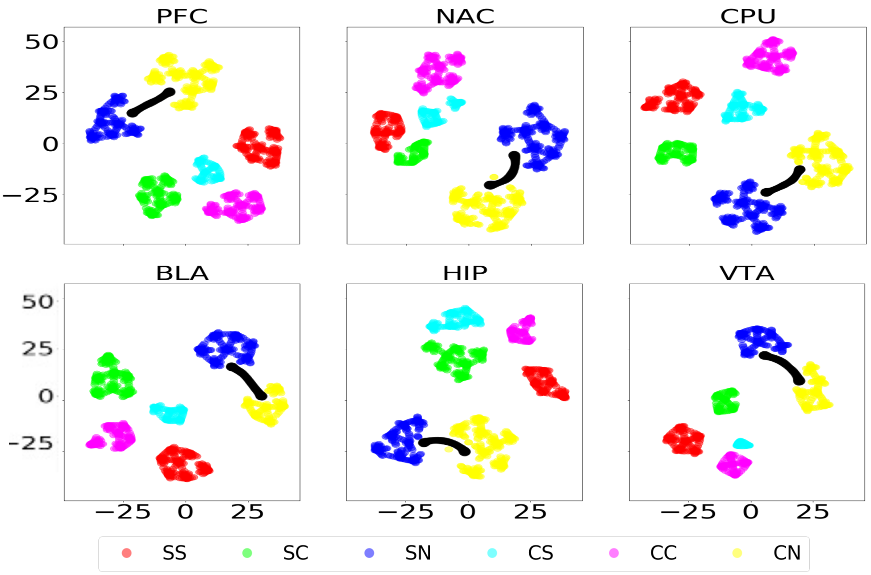

Data augmentation using linear interpolation and its validation through t-SNE validation

(**a**) Data augmentation using linear interpolation. (**b**) t-SNE validation for augmented data. Black color indicates transition curve from SN to CN.

Supplementary Figure 2

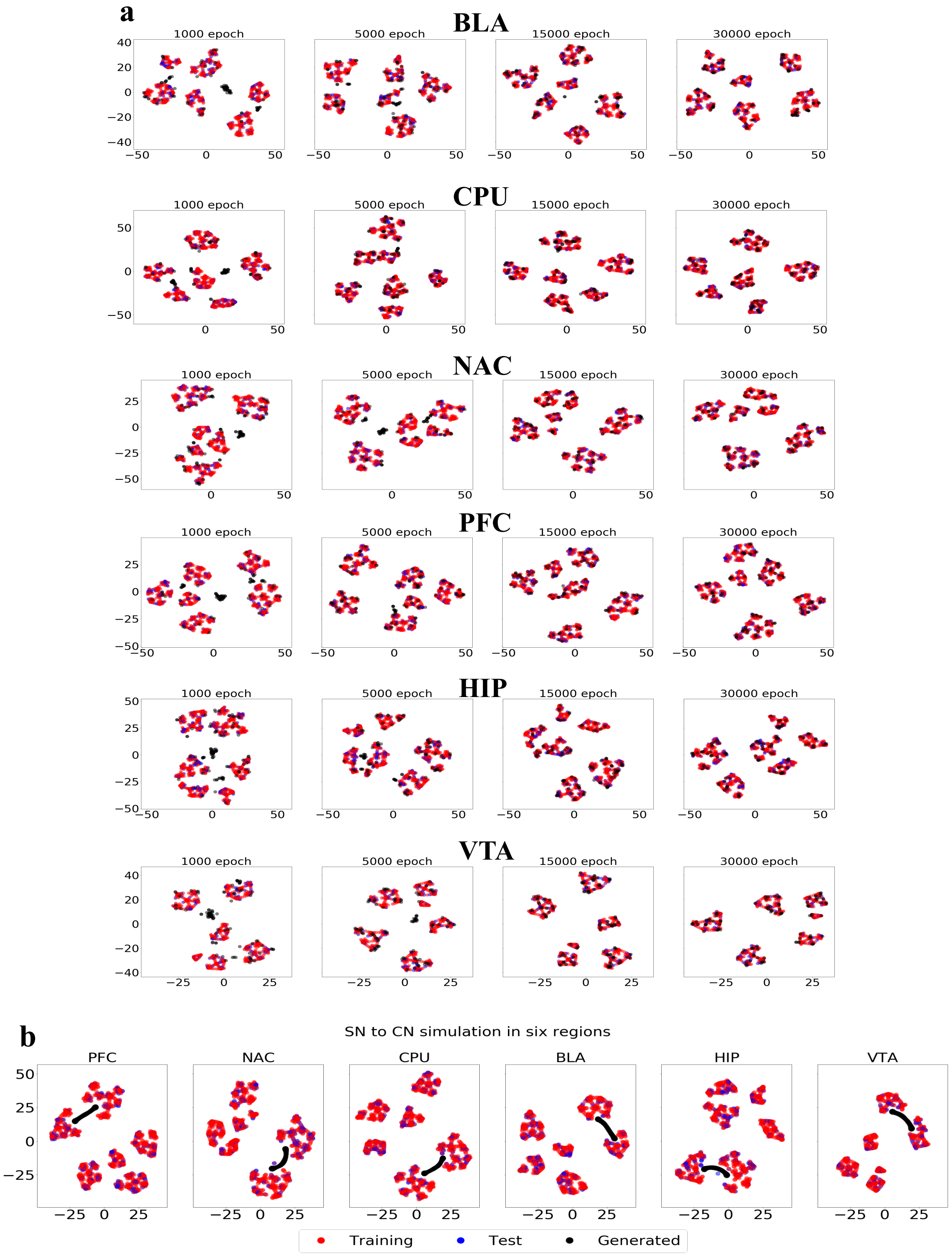

Averaged gene expression simulation using GAN and its validation using t-SNE (**a**) Clustering between training and test and generated data using t-SNE. As epoch goes by, we observed remarkably well overlapped cluster by group**.** Red, blue and black dots represent training, test and generated data, respectively. (**b**) A delta vector of SN to CN was constructed using 10 nearest latent vectors based on the Pearson's correlation among 35,000 fakes. Constructed delta used to generate gene expression data of addiction progress – 100 dots between SN to CN.

Supplementary Figure 3

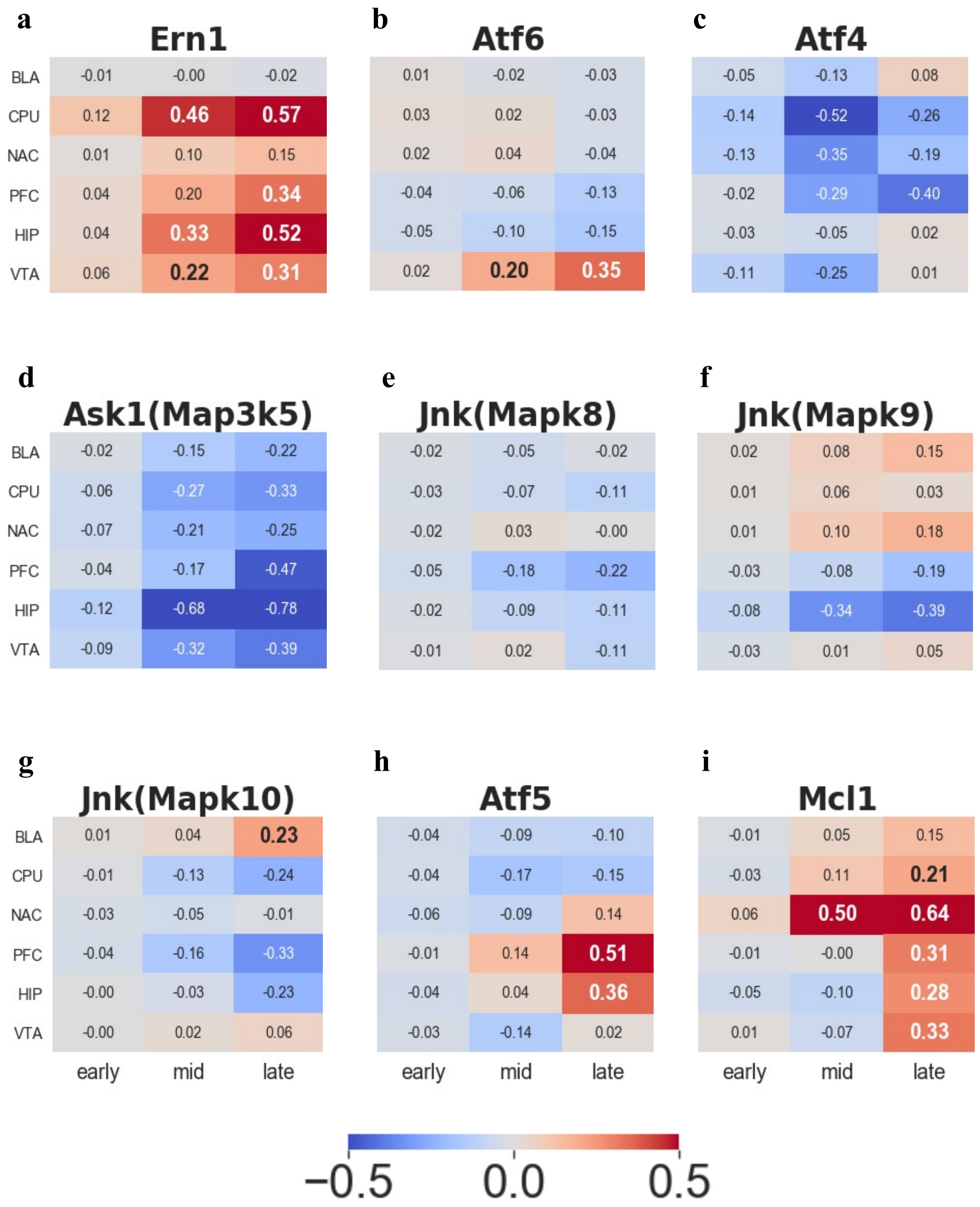

Log2FC Trend for UPR and apoptosis-related gene- heatmap.
(**a**) Inositol-requiring enzyme 1 a (*Ire1*a or *Ern1*) (**b**) Activating transcription factor 6 (*Atf6*). **a** and **b** are both ER stress sensor. (**c**) Activating transcription factor 4 (*Atf4*) (**d**) Apoptosis signal-regulated kinase 1 (*Ask1*) (**e**) Mitogen-activated protein kinase 8 (*Mapk8*) (**f**) Mitogen-activated protein kinase 9 (*Mapk9*) (**g**) Mitogen-activated protein kinase 10 (*Mapk10*) (**h**) Activating transcription factor 5 (*Atf5*) (**i**) Induced myeloid leukemia cell differentiation protein (*Mcl1*)

Supplementary Figure 4

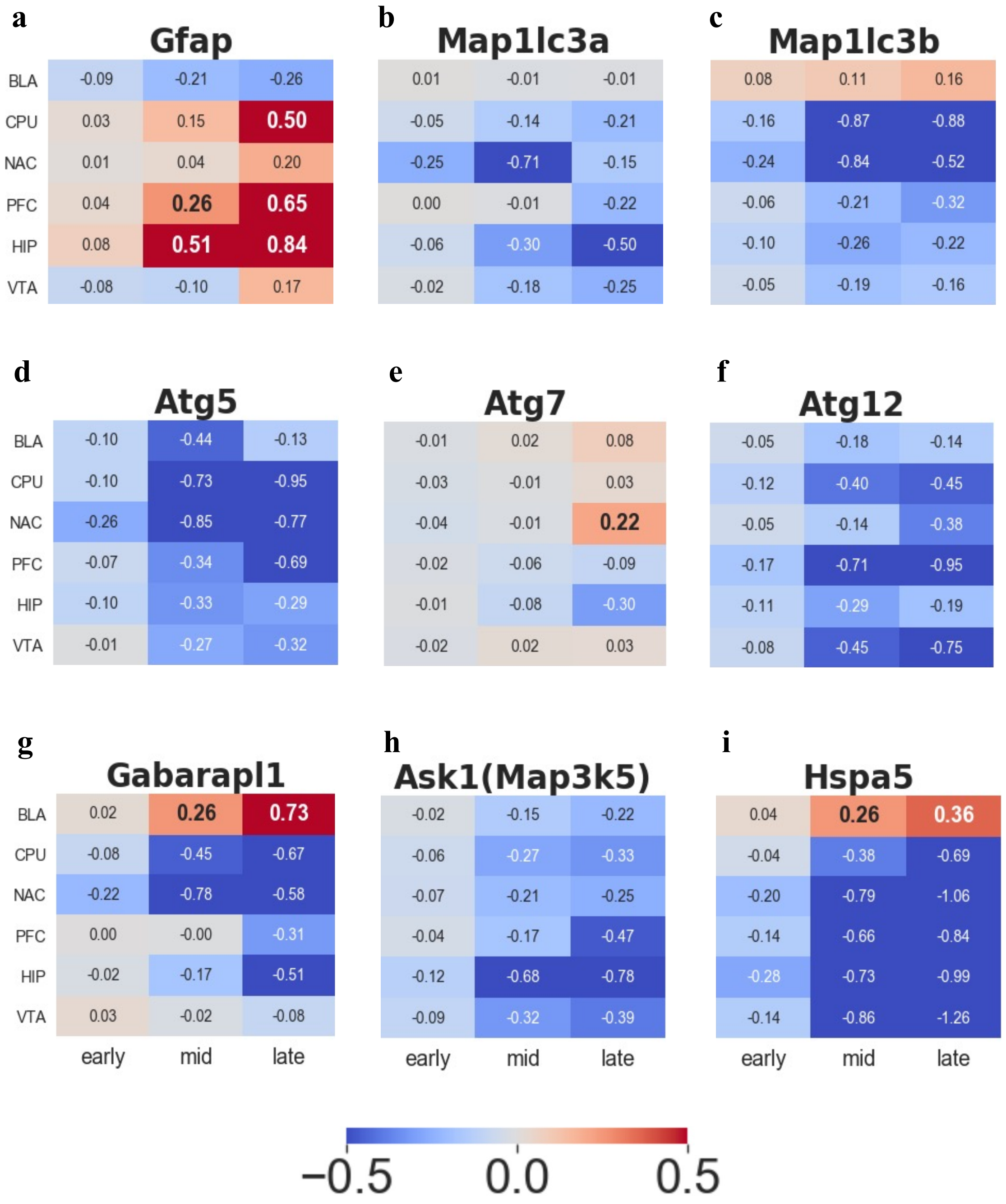

Log2FC Trend for autophagy and reactive astrocyte related gene.
(**a**) Glial fibrillary acidic protein (*Gfap*) (**b**) Microtuble-associated proteins 1A/1B light chain 3A (*Map1lc3a*) (**c**) Microtuble-associated proteins 1A/1B light chain 3B (*Map1lc3b*) (**d**) Autophagy related 5 (*Atg5*) (**e**) Autophagy related 7 (*Atg7*) (**f**) Autophagy related 12 (*Atg12*) (**g**) Gamma-aminobutyric acid receptor-associated protein-like 1 (*Gabarapl1*) (**h**) Apoptosis signal-regulated kinase 1 (*Ask1*) (**i**) heat shock 70 kDa protein 5 (*Hspa5*)

Supplementary Figure 5

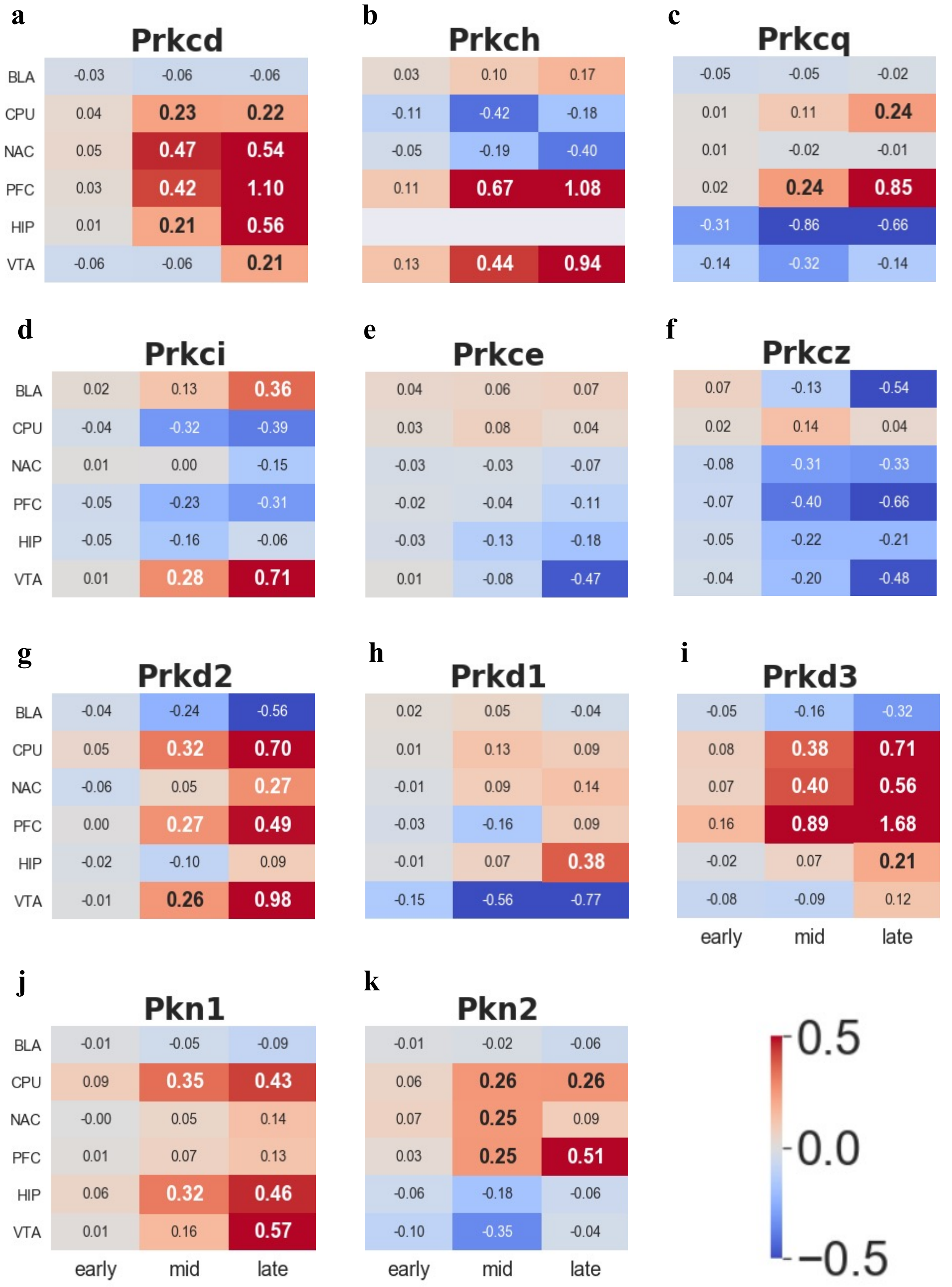

Log2FC Trend for *Pkc* family.
(**a**) Protein kinase C delta type (*Prkcd*) (**b**) Protein kinase C eta type (*Prkch*)
(**c**) Protein kinase C theta (*Prkcq*) (**d**) Protein kinase C iota (*Prkci*)
(**e**) Protein kinase C epsilon (*Prkce*) (**f**) Protein kinase C zeta (*Prkcz*)
(**g**) Protein kinase D2 (*Prkd2*) (**h**) Protein Kinase D1 (*Prkd1*) (**i**) Protein kinase D3 (*Prkd3*)
(**j**) Protein kinase N1 (*Pkn1*) (**k**) Protein kinase N2 (*Pkn2*)

Supplementary Figure 6

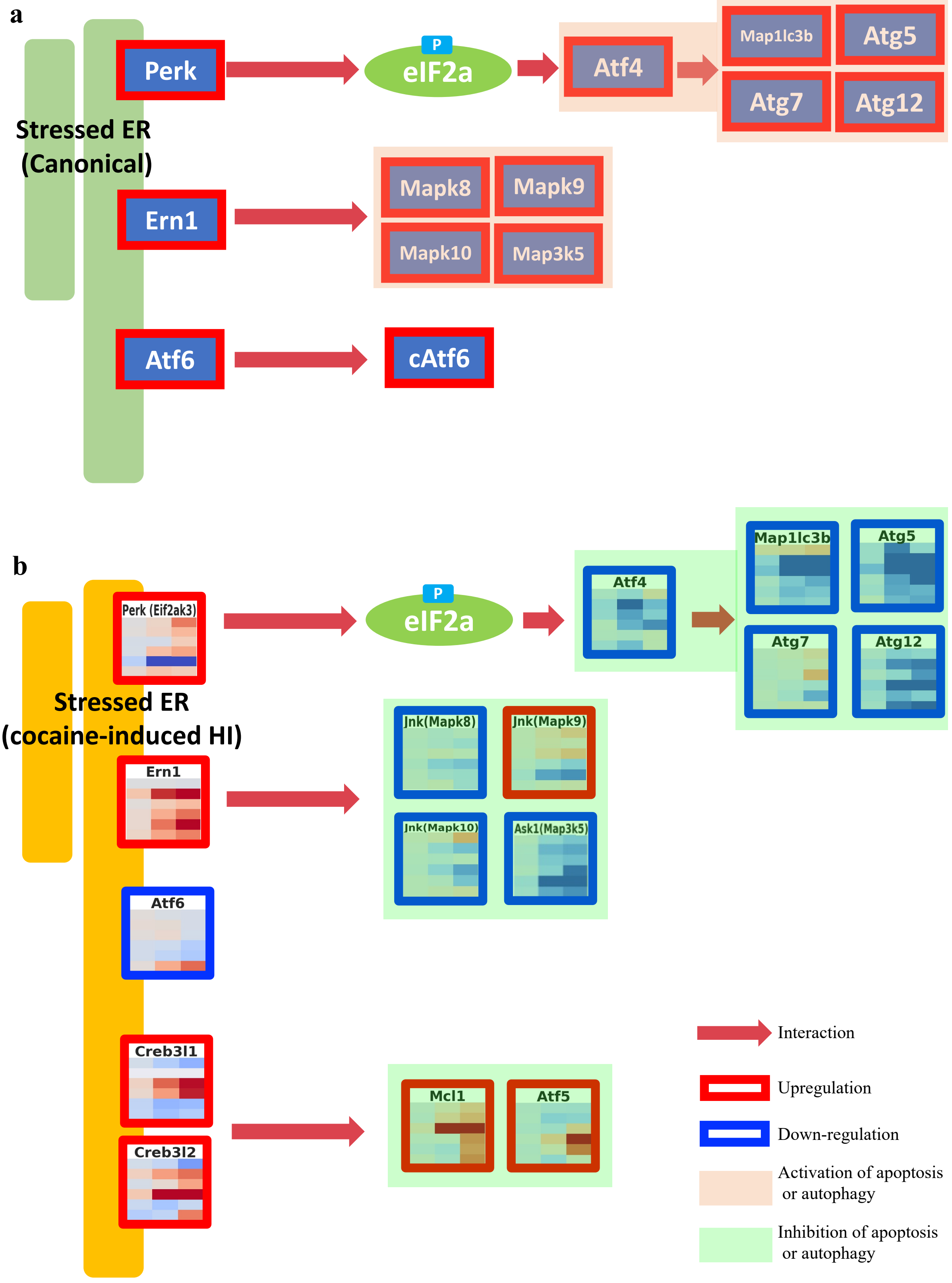

Description of ER stress pathway

(a) Canonical ER stress pathway **(b)** ER stress pathway induced by cocaine-induced HI

Supplementary Figure 7

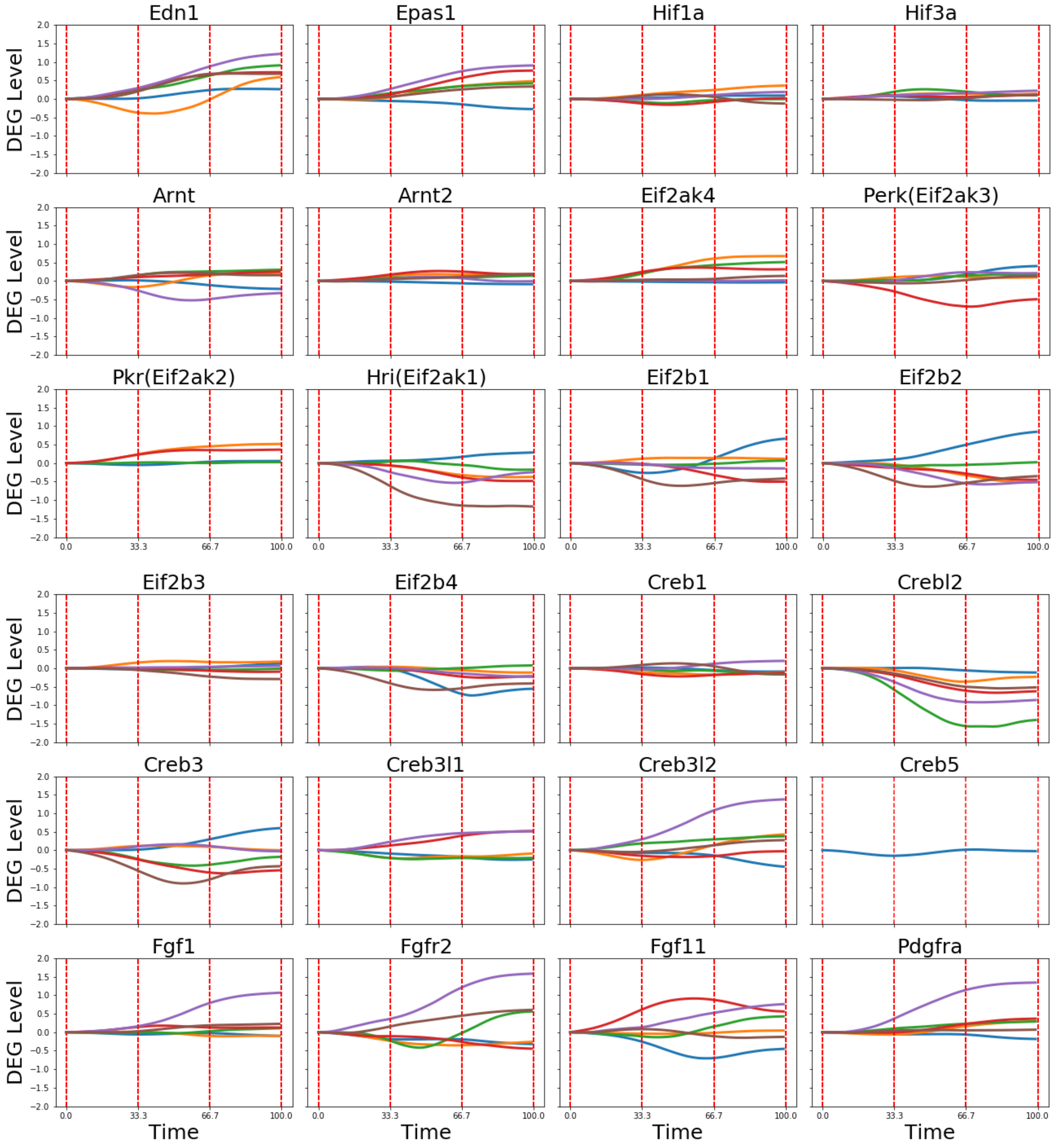

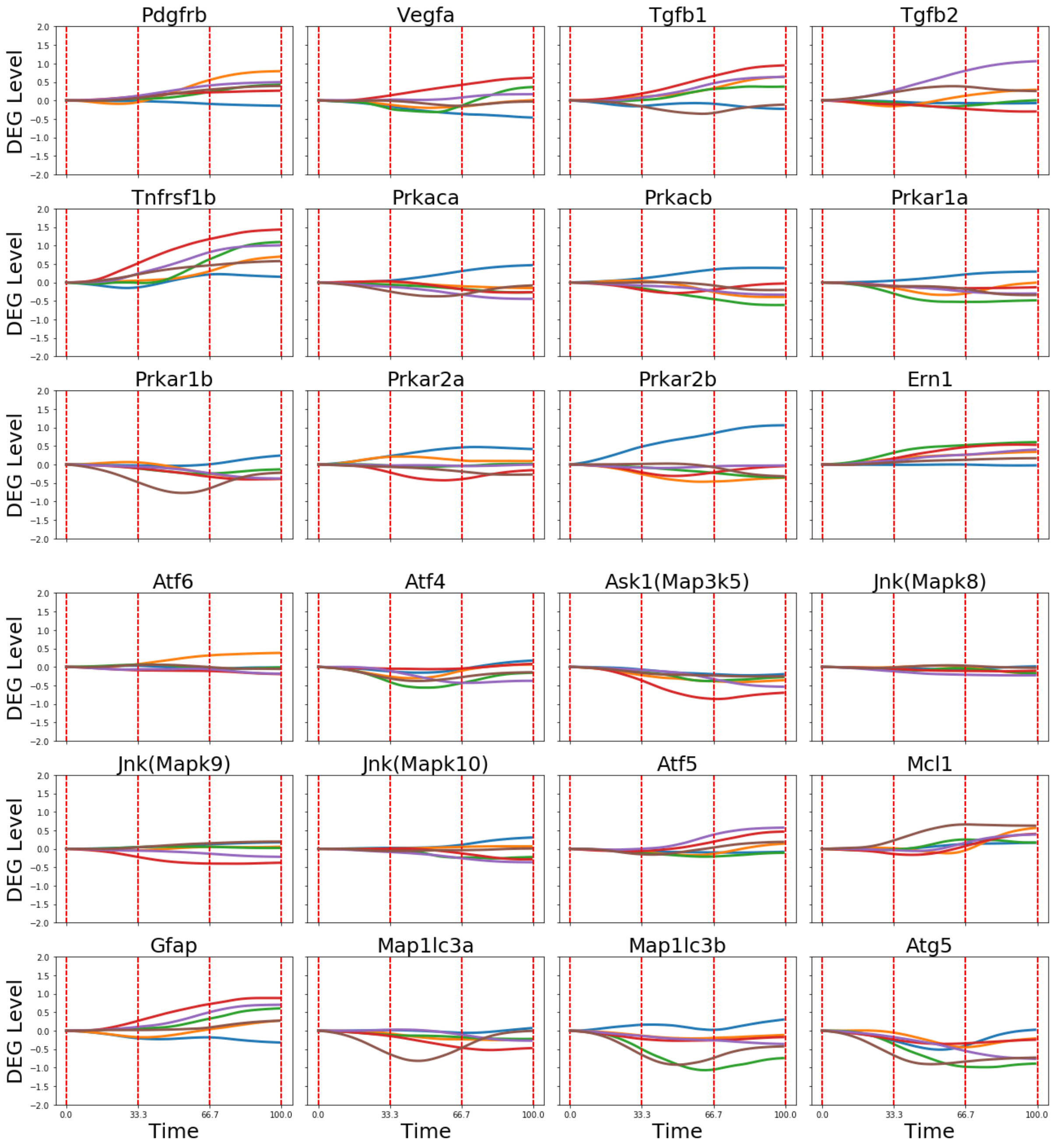
Pdgfrb
Vegfa
Tgfb1
Tgfb2
Tnfrsf1b
Prkaca
Prkacb
Prkar1a
Prkar1b
Prkar2a
Prkar2b
Ern1
Atf6
Atf4
Ask1(Map3k5)
Jnk(Mapk8)
Jnk(Mapk9)
Jnk(Mapk10)
Atf5
Mcl1
Gfap
Map1lc3a
Map1lc3b
Atg5
DEG Level
2.0
1.5
1.0
0.5
0.0
-0.5
-1.0
-1.5
-2.0
0.0
33.3
66.7
100.0
Time

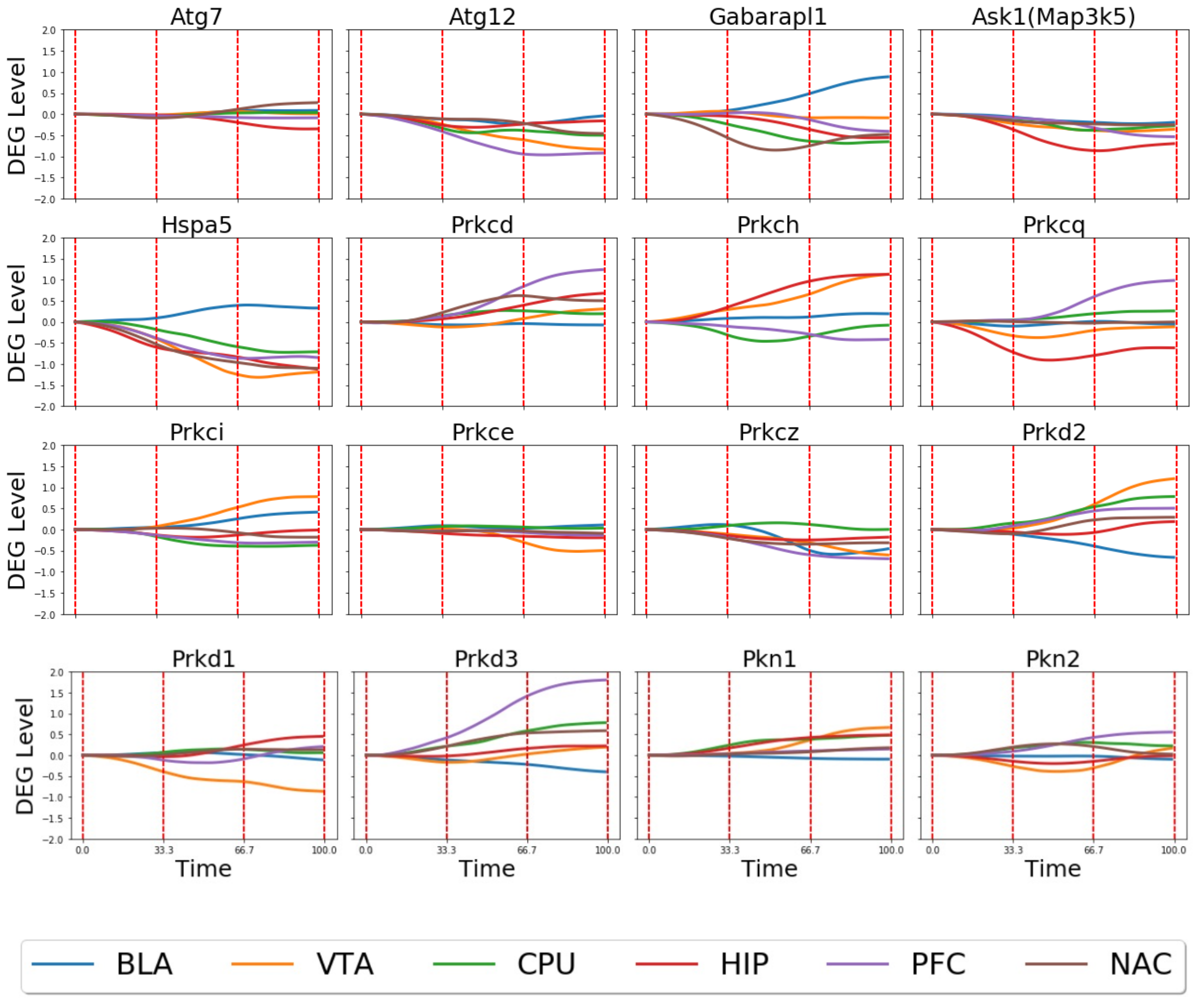

Log2FC trend of continuous mRNA profiles
Red dotted lines indicate starting and end points of each time intervals

Supplementary table1

**Hyperparmeter**	**Value**
**z_dim** (dimention of noise vector)	**100**
**Lambda**	**10**
**L_r** (learning rate)	**0.1e-5**
**G** (The number of nodes for generator)	**800**
**D** (The number of nodes for discriminator)	**400**
**batch_size**	**32**
**R_threshold** (Pearson correlation)	**0.95**
